# Psilocybin-assisted psychotherapy for Parkinson's disease without depression: A case-report

**DOI:** 10.1177/1877718X241312604

**Published:** 2025-02-02

**Authors:** Vanessa Fleury, Emilie Tomkova, Sabina Catalano Chiuvé, Louise Penzenstadler

**Affiliations:** 1 Department of Neurology, Geneva University Hospital, Geneva, Switzerland; 2 Faculty of Medicine, University of Geneva, Geneva, Switzerland; 3 Department of Psychiatry, Geneva University Hospital, Geneva, Switzerland

**Keywords:** Psilocybin, Parkinson's disease, quality of life, resilience, acceptance

## Abstract

**Background:**

Psychedelic assisted psychotherapy (PAP) can improve treatment-resistant depression. Its usefulness in Parkinson's disease (PD) is unknown. PD patients may have problems adjusting to their chronic progressive neurological disease. A change from emotional avoidance to acceptance has been reported following psilocybin administration in patients with treatment-resistant depression.

**Objective:**

To report for the first time the effect of psilocybin in a PD patient.

**Methods:**

A non-depressed 43-year-old female with a 2-year history of PD presented with difficulty adjusting to PD, anxious ruminations and pessimism. The patient declined an increase in dopaminergic medication or the introduction of an anxiolytic. Therapeutic patient education was not beneficial. The patient received four sessions of high-dose PAP within one year. Neurological and psychiatric assessments were performed before and at one year follow-up using qualitative interviews and quantitative assessment of motor status, dispositional optimism, depression, anxiety, apathy, and well-being.

**Results:**

PAP was well tolerated. It significantly improved the patient's overall pessimistic outlook on her future and decreased her anxious ruminations and worries about potential handicap due to PD. Her general well-being improved, as well as all psychometric scores except for the apathy scale. Motor status remained unchanged. Better acceptance of PD allowed her to accept pharmacological treatment adjustment.

**Conclusions:**

PAP could be a safe and useful treatment for PD patients with dispositional pessimism and difficulties accepting their disease by promoting profound decentration from habitual thoughts and emotions, improving mood and PD acceptance. Randomized, controlled studies are needed to confirm this result.

## Plain language summary

This study explored the effects of psilocybin-assisted psychotherapy (PAP) on a 43-year-old woman with Parkinson's disease (PD), who struggled to accept her diagnosis, leading to anxious ruminations and pessimism. Treatment adjustments were considered but were either ineffective or declined by the patient. The patient received four high-dose PAP sessions over a year. The therapy was well-tolerated, reduced her anxious thoughts, and improved her outlook on the future and general well-being. The patient accepted medication adjustments. The study suggests PAP might help PD patients with high dispositional pessimism and acceptance issues, though more research is needed to confirm these findings.

## Introduction

In combination with psychotherapy, the psychedelic psilocybin^1^ sustainably and safely improves depressive symptoms and anxiety in treatment-resistant depression.^2–4^ Psilocybin may also be effective in substance use disorder,^5,6^ and obsessive-compulsive disorder.^7^

Parkinson's disease (PD) presents with motor and non-motor symptoms that greatly impact patient's quality of life (QoL).^8^ PD is emotionally distressing for many patients and disease acceptance may be difficult.^9^ Lower disease acceptance represents an obstacle to healthy adjustment, and leads to decreased life satisfaction, depression, anxiety, drug adherence and poorer QoL.^10–12^

The benefits of psilocybin in neurodegenerative disorders such as PD and Alzheimer's disease are not yet known.^13,14^ An open-label pilot study is underway to assess the safety and efficacy of psilocybin for depression in PD patients (NCT04932434).

Here we report the first case of a PD patient who was successfully treated with psilocybin-assisted psychotherapy (PAP)^15^ which improved patient's pessimism, well-being, PD acceptance and medication adherence.

## Case report

The patient was a right-handed 43-year-old female who presented with a 2-year history of PD predominantly in her right arm. She had no prior medical history other than previous tobacco consumption (5 packs year). She had no personal or family psychiatric history. In her youth she had occasionally consumed marijuana as a recreational drug. She was well-integrated socially. Pramipexole ER 1.875 mg partially controlled her symptoms. The patient retained functional disability in her dominant hand. She had no signs of depression but exhibited anxious ruminations and worries about her future and potential handicap due to PD leading to short periods of low mood and an overall pessimistic outlook. Her MDS-UPDRS^16^ motor score was 21/132, with a moderate akinetic rigid syndrome predominating in her right arm. Her neuropsychiatric status was normal with good motivation (Starkstein^17^ = 1/42), no anxiety (STAI-state^18^ = 24/80) nor depression (BDI-II^19^ = 9/63, HDRS^20^ = 4/52) and a normal neuropsychiatric fluctuation scale (NFS) score^21^ (Table 1). The Revised Life Orientation Test^22^ (LOT-R) assessing dispositional optimism, was 10/24, indicating high pessimism. Pramipexole was increased but was later tapered due to poor tolerance. The patient declined therapy with safinamide, levodopa or an anxiolytic. Therapeutic Patient Education with our specialized Parkinson nurse was not beneficial. The patient therefore sought alternative approaches.^23^ In this context, she read about psilocybin therapeutic benefits. She saw psychedelics as shamanic plants that had been used for millennia, offering a holistic, natural approach and a complement to the use of her PD medication. The patient agreed to the suggestion to try PAP and was reassured by the fact that psilocybin was to be administrated under medical supervision in a structured PAP program.^15^

**Table 1.** Patient's neurological and psychiatric characteristics before and one-year after psilocybin-assisted psychotherapy.

	Before treatment	1 year follow-up
Life-Orientation Test (LOT-R) (/24)	**10**	20
State-Trait Anxiety Inventory (STAI)		
STAI-state (/80)	24	22
STAI-trait (/80)	29	25
Beck Depression Inventory (BDI-II) (/63)	9	1
Hamilton Depression Rating Scale (HDRS) (/52)	4	0
Starkstein apathy score (/42)	1	10
Neuropsychiatric fluctuation scale (NFS)		
Items ON (/30)	25	28
Items OFF (/30)	3	0
PD-Non Motor Symptoms scale (/40)	3	4
MDS-UPDRS III (/132)	21	20
Levodopa equivalent dose (mg)	187.5	425.0

A Starkstein apathy score of ≥14/42, an anxiety State-Trait Anxiety Inventory (STAI) score ≥ 50/80 for State and ≥48/80 for Trait, a depression Beck Depression Inventory (BDI) score ≥ 21/63, a Hamilton Depression Rating Scale (HDRS) score ≥ 8/52, a Life-Orientation Test (LOT-R) score ≤ 13/24 were pathological cut-off values. Pathological scores are shown in bold.

The Neuropsychiatric fluctuation scale (NFS) explores well-being, self-confidence and energy level.

Authorization for the treatment was obtained from the Federal Office of Public Health. The patient received four high-dose sessions of PAP within one year (25 mg for the first three sessions and 30 mg for the fourth session). Psilocybin doses were chosen in line with current trials, typically ranging from 20 to 30 mg per session.^24–27^ The sessions took place in an outpatient setting at 10–18 week intervals. Each session included psychotherapy with 1) a preparation meeting to establish the therapeutic alliance, prepare the patient for the psychedelic experience and set treatment goals; 2) an integration consultation the day after psilocybin administration to help the patient to make sense of the subjective psychedelic experience and how to use it to benefit her psychotherapeutic work; 3) a follow-up session one month afterwards to assess the long-term effects, provide additional support and evaluate the therapeutic process.

All psilocybin sessions were closely monitored by an experienced team of psychiatrists and nurses. During treatment, no serious adverse effects were reported. The patient experienced an altered state of consciousness characterized by profound alterations in mood and perception of reality and the sense of self and meaning.^28^ The occurrence and magnitude of the psilocybin-induced “oceanic boundlessness” experience (i.e., feeling of ecstatic self-expansion, unity and transcendence) was high. The patient did not experience anxious ego-dissolution.

One year later, the patient demonstrated significant improvement in her well-being. She reported reduced anxious ruminations and a more optimistic view on life. This was reflected in the improved LOT-R score of 20/24, indicating high optimism. According to the patient, psilocybin also improved her sleep and smell, and helped her to better integrate her illness. “I have accepted that PD is part of me”. The patient was able to better prioritize her personal well-being and to appreciate the present moment. All psychometric scores, although non-pathological before treatment, improved except for the Apathy scale which remained within the normal range (Table 1). The patient remained very active. Regarding her motor symptoms, she described a subjective improvement after each session. Her MDS-UPDRS motor score was stable at one-year follow-up (Table 1). The improvement in her disease experience and acceptance subsequently led the patient to agree to increase dopaminergic treatment. At 1-year follow-up, she was taking pramipexole ER 2.25 mg, safinamide 1 mg and levodopa 62.5 mg/day.

### Ethicss

Written informed consent was obtained from the patient for publication of this case report. Ethical approval was not required for a case report in accordance with the local Geneva Ethics Committee.

## Discussion

PAP enabled this PD patient to shift her point of view and to have a more positive vision of her future. It enabled her to initiate the PD acceptance process and to accept an increase in dopaminergic therapy. Her disease management moved from a predominantly physical management to a deeper emotional integration, fostering improved well-being.

PAP facilitates a profound and potentially transformative psychological experience.^28^ High level of psilocybin-induced oceanic boundlessness and low dread of ego dissolution correlated with positive long-term clinical outcomes.^29,30^ As our patient presented with existential anxiety and ruminations, shifting focus from the self to the transcendent (such as family, community, or the universe) prompted a revision of her own reality.^31–33^ Decentration (i.e., the ability to view thoughts and emotions as objective mental events rather than personal identifiers),^34^ was another key phenomenon. The synergistic effect of psychedelics helps patients detach from their identity and personal concerns, encouraging a more objective and adaptative perspective on their mental experiences.

Neurobiologically, psilocybin improves cognitive flexibility, creative cognition, and emotional processing.^35–37^ PAP can unlock difficult-to-access memories and emotions,^38^ reduce avoidance and increase acceptance of such emotions^39^ and facilitate cognitive restructuring. Psilocybin increases cortical entropy^40^ and desynchronizes human brain.^1,41^ We hypothesized that long-term cerebral changes induced by PAP could improve emotional and executive networks that are already dysfunctional in PD, improving resilience and disease acceptance.

Psilocybin is a serotonin receptor agonist and has a positive effect on mood.^2,3,25,42,43^ Our patient had high dispositional pessimism and PAP helped her to develop higher optimism. This is an important therapeutic effect, as higher dispositional optimism predicts a more satisfactory QoL and lower emotional distress.^44^

Regarding limitations, a case report is not controlled and does not allow the generalization of the current results to a larger population.

In conclusion, psilocybin facilitates psychotherapy and could be a safe and useful interdisciplinary strategy to support PD patients, particularly those with high dispositional pessimism and difficulties with disease adjustment and acceptance. Randomized, controlled studies are needed to confirm this result. Their implementation should be facilitated by ongoing efforts within the United Nations Office on Drugs and Crime to reclassify psilocybin as non-addictive.^45,46^
